# Chronic, but not acute, treatment with GLP‐2 and GIP in combination increases glucose absorption in the isolated vascularly perfused rat intestine

**DOI:** 10.14814/phy2.71069

**Published:** 2026-08-25

**Authors:** Katrine D. Galsgaard, Tyler A. Cookson, Ida M. Modvig, Valentina Abba, Samuel A. J. Trammell, Mette M. Rosenkilde, Lærke S. Gasbjerg, Charlotte M. Sørensen, Bolette Hartmann, Jens J. Holst

**Affiliations:** ^1^ Department of Biomedical Sciences, Faculty of Health and Medical Sciences University of Copenhagen Copenhagen Denmark; ^2^ Novo Nordisk Foundation Center for Basic Metabolic Research, Faculty of Health and Medical Sciences University of Copenhagen Copenhagen Denmark

**Keywords:** glucagon‐like peptide‐2, glucose‐dependent insulinotropic polypeptide, G‐protein‐coupled receptor, intestinal blood flow, nutrient absorption, perfused rat intestine

## Abstract

The gut hormones, glucagon‐like peptide‐2 (GLP‐2) and glucose‐dependent insulinotropic polypeptide (GIP), may be important regulators of intestinal nutrient absorption. Using the isolated vascularly perfused rat intestine of male rats, we investigated the chronic and subacute (injections 6 days and 2 h, respectively, prior to perfusion) effects of a long‐acting GLP‐2‐receptor (GLP‐2R) agonist, a long‐acting GIP‐receptor (GIPR) agonist, and their combination on nutrient absorption. Potential acute effects were studied by administering native GLP‐2, GIP, and GLP‐2 + GIP intravascularly during the perfusion. During the perfusions investigating the effects of GLP‐2, GIP, and the long‐acting receptor agonists, the arterial perfusion flow was kept constant. In separate experiments, the dependency of superior mesenteric artery flow on nutrient absorption was studied by changing the arterial perfusion flow. The effects of the long‐acting receptor agonists on superior mesenteric blood flow were additionally assessed in vivo using a transit time flow probe. Chronic GLP‐2R agonist treatment alone increased intestinal size and glucose absorption, and combination with GIPR agonist treatment did not further increase intestinal size and glucose absorption. None of the treatments affected the absorption of short‐ and medium‐chain fatty acids or amino acids. Nutrient absorption increased with the vascular perfusion flow rate. The GIPR agonist increased superior mesenteric artery blood flow alone and when combined with the GLP‐2R agonist, whereas the GLP‐2R agonist had no significant effect. Our results suggest that chronic GLP‐2R agonism may increase glucose absorption by increasing intestinal size, and that increasing intestinal blood flow may increase nutrient absorption.

## Graphical abstract

Chronic GLP‐2 + GIP treatment increases glucose absorption.

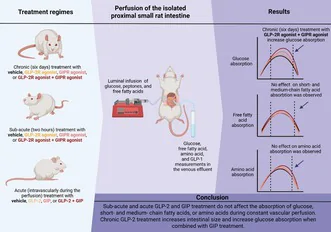

## 1 INTRODUCTION

During a meal, gut hormones, including glucagon‐like peptide‐1 (GLP‐1), glucagon‐like peptide‐2 (GLP‐2), glucose‐dependent insulinotropic polypeptide (GIP), and peptide YY, are released from the enteroendocrine cells. In concert with the enteric nervous system, these hormones are thought to regulate postprandial nutrient metabolism, appetite, and splanchnic blood flow (Bergmann et al., 2019; Koffert et al., 2017; Okazaki et al., 1986), which increase after a meal (Bremholm et al., 2009). Intestinal nutrient absorption is facilitated by a variety of specialized nutrient transporters, including sodium/glucose co‐transporter 1 (SGLT1), the basolateral glucose transporter 2 (GLUT2), and peptide transporter 1 (PEPT1) transporting glucose and di‐/tripeptides, respectively, and brush border enzymes (e.g., lactase, sucrose/isomaltase, and aminopeptidases) present in the intestinal mucosa. Together, the nutrient transporters and brush border enzymes ensure digestion and transport of nutrients from the intestinal lumen to the blood.

GLP‐2 administration promotes expansion of the intestinal mucosa (through crypt cell proliferation and reduced enterocyte apoptosis) (Estall & Drucker, 2003; Høyerup et al., 2013; Jeppesen et al., 2005), increases intestinal blood flow in vivo (Bremholm et al., 2009; Bremholm et al., 2011), and has been reported to increase expression and activity of SGLT1, GLUT2, PEPT1 (Au et al., 2002; Cheeseman, 1997; Hu et al., 2010; Ramsanahie et al., 2003), and certain brush border enzymes (Brubaker et al., 1997; Kitchen et al., 2000; Petersen et al., 2001; Petersen et al., 2002). Together this may result in improved barrier function (Cani et al., 2009) and increased intestinal absorption of fluids, nutrients, and electrolytes (Jeppesen et al., 2001; Jeppesen et al., 2009). These well‐established effects of GLP‐2 have inspired the development of long‐acting GLP‐2 analogous, teduglutide and glepaglutide, designed for the treatment of intestinal insufficiency (Jeppesen et al., 2011; Jeppesen et al., 2012; Jeppesen et al., 2025).

GIP administration has been reported to increase the activity of SGLT1 and PEPT1 (Coon et al., 2013; Coon et al., 2015; Singh et al., 2008), as well as intestinal blood flow (Fara & Salazar, 1978; Honka et al., 2017; Koffert et al., 2017; Kogire et al., 1988; Kogire et al., 1992; Rasmussen et al., 2025), and thus GIP may also increase intestinal nutrient absorption. The specific effects of GIP on intestinal parameters, such as digestion, absorption, and blood flow, have been overshadowed by its insulinotropic effects, but with the recent development of GLP‐1/GIP receptor (Willard et al., 2020) and GIP/GLP‐2 receptor (Gabe et al., 2022) co‐agonists for the treatment of obesity (Pálsson et al., 2024) and osteoporosis (Gabe et al., 2022), the interest in GIP actions has been revived.

We hypothesized that GLP‐2 and GIP combined might exert a synergistic stimulatory effect on intestinal nutrient absorption as well as intestinal blood flow. We tested these hypotheses using long‐acting GLP‐2R and GIPR agonists and native GLP‐2 and GIP, and intestinal absorption was investigated during three different treatment regimens: (I) a chronic (6 days) treatment allowing structural changes (growth effects) in the intestine to take place, (II) a sub‐acute (2 h) treatment allowing time for synthesis and translocation of transporters and brush border enzymes, and (III) an acute (intravascularly during the perfusion) treatment in theory allowing only translocation of transporters to the apical and basolateral enterocyte membranes. Establishing the effects of GLP‐2, GIP, and GLP‐2 + GIP on nutrient absorption capacity is of interest in the development of therapeutics for intestinal insufficiency.

## 2 METHODS

### 2.1 Animals and ethical considerations

All animal studies were approved by the Danish Animal Experiments Inspectorate (2020‐15‐0201‐00547 and 2023‐15‐0201‐01408) and the local ethical committee (Department of Experimental Medicine). Male Sprague–Dawley rats, weighing approximately 350 g, were obtained from Janvier Labs (Le Genest‐Saint‐Isle, France). All rats were fed ad libitum with regular chow (Scientific diets, SAFE^®^ D30). Rats were housed two to four animals per cage in a 12:12‐h light–dark cycle and allowed at least 1 week of acclimatization prior to experiments.

### 2.2 Vascular perfusion of the isolated rat small intestine

Non‐fasted rats were anesthetized by subcutaneous injection of 0.3 mL/100 g Hypnorm/Midazolam (Hypnorm: 0.315 mg/mL fentanyl citrate and 10 mg/mL fluanisone, Midazolam: 5 mg/mL) with the exception of the perfusions in which the arterial perfusion flow was varied; here the rats were anesthetized by subcutaneous injection of ketamine 75 mg/kg and Dexmedetomidine 0.75 mg/kg (0.4 mL/100 g) diluted in sterile saline. Upon sedation the intestines were isolated and perfused as described in detail elsewhere (Kuhre & Holst, 2019). The small intestines were perfused for 25 min prior to beginning the experimental protocol. Protocols began with a 10 min baseline period of luminal 0.9% saline infusion, followed by intraluminal administration of a 10% w/v glucose, 25 mg/mL peptone, and 50 mM short‐ and medium‐chain fatty acid solution. The fatty acids included: sodium acetate (C2:0), sodium propionate (C3:0), sodium butyrate (C4:0), caproic acid (C6:0), and caprylic acid (C8:0). These were mixed in equal concentrations to yield a net concentration of 50 mM fatty acids. Caproic acid and caprylic acid were first dissolved in DMSO. The final concentration of DMSO in the solution did not exceed 1%. A 5‐min intravascular infusion of 10 nM bombesin (BBS, Sigma‐Aldrich, cat. no. B4272) or 50 mM KCl was included at the end of all experiments as a positive control evaluating glucagon‐like peptide 1 (GLP‐1) secretion. All solutions were pre‐heated prior to administration. Luminal solutions were administered at a rate of 0.25 mL/min using a syringe pump. The rate was increased to 0.5 mL/min for 2 min each time intraluminal solutions were changed and then returned to 0.25 mL/min until the next change. Vascular flow and perfusion pressure were monitored throughout the experiment. Following the perfusion, the perfused intestinal segments were weighed, length recorded, and biopsies were obtained from the proximal and distal part of the perfused intestinal segments. Biopsies were frozen on dry ice, submerged in RNAlater (Sigma Aldrich, cat no. R0901), or submerged in formaldehyde (Hounisen, cat no. 1000.0020).

### 2.3 Chronic treatment with GLP‐2R and GIPR agonists

Rats were given subcutaneous injections (2 mL/kg) once daily for 6 days with either (1) vehicle (69 mM phosphate buffer pH 7.5 + 0.1% Haemaccel), (2) GLP‐2R agonist (CP103 (Gadgaard et al., 2023), kindly provided by Bainan Biotech, Copenhagen, 8 mg/kg dissolved in vehicle), (3) GIPR agonist (BB138 (Tordrup et al., 2025), kindly provided by Bainan Biotech, Copenhagen, 9.6 mg/kg, to achieve equimolar doses of the GLP‐2R agonist and GIPR agonist, dissolved in vehicle), or (4) GLP‐2R agonist + GIPR agonist. These GLP‐2R and GIPR agonists were selected for their high potency on the rat receptors and duration of action (Gadgaard et al., 2023; Tordrup et al., 2025). Groups 1–3 were given a second vehicle injection to account for the two injections in group 4. The rats were weighed daily during the treatment period, and blood glucose was measured from the tail vein prior to isolation and perfusion of the intestine.

### 2.4 Subacute treatment with GLP‐2R and GIPR agonists

In these experiments, rats were treated as described above for groups 1–4; however, the rats received only single injections 2 h prior to perfusion of the intestine.

### 2.5 Acute treatment with native GLP‐2 and GIP

In these experiments, native human GLP‐2(1‐33) (Bachem, Bubendorf, Switzerland, H‐7722) and human GIP(1‐42) (Custom made, PolyPeptide group, Strasbourg, France) were dissolved in a 50 nM sodium hydrogen carbonate buffer, pH 8.5, containing in addition 0.5% human serum albumin and NaCl 9 mg/mL and prepared by the Capital Region Pharmacy (Herlev, Denmark). Both peptide solutions had a purity above 97%. GLP‐2, GIP, or GLP‐2 + GIP were infused intravascularly at a rate of 0.375 mL/min reaching a final concentration of 10 nM each. The infusion began 15 min after the intraluminal macronutrient infusion was started and continued for 20 min during the intraluminal macronutrient infusion. In the vehicle group, perfusion buffer was administered intravascularly at the same infusion rate.

### 2.6 Alterations in vascular perfusion flow

In these experiments the intravascular perfusion flow was changed from the standard 7.5 mL/min to 10 mL/min and then to 3 mL/min to return to 7.5 mL/min for the remainder of the perfusion. The intraluminal macronutrient infusion was kept constant as described above.

### 2.7 In vitro pharmacological characterization

Transfections and tissue culture: COS‐7 cells (ATCC, Virginia, USA) were cultured at 10% CO_2_ and 37°C in DMEM 1885 supplemented with 10% FBS, 2 mM glutamine, 180 units × mL^−1^ penicillin and 45 μg × mL^−1^ streptomycin. Transient transfection with the rat GIP receptor or GLP‐2 receptor was performed using the calcium phosphate precipitation method with the addition of chloroquine (Thermo Scientific, cas. no. 50‐63‐5) (Kissow et al., 2012). cAMP assay: Transiently transfected COS‐7 cells were seeded out in white 96‐well plates at a density of 3 × 10^4^ cells per well. The day after, the cells were washed twice with HEPES‐buffered saline (HBS) buffer and incubated with HBS and 1 mM IBMX (Sigma‐Aldrich, cat no. 5879) for 30 min at 37°C. The hormones and receptor agonists were added and incubated for 30 min at 37°C. The HitHunterTM cAMP XS assay (DiscoveRx, Herlev, Denmark, cat. no. 90‐0075SM2) was carried out according to the manufacturer's instructions.

### 2.8 Histology

Biopsies were kept in formaldehyde for 24 h and then transferred to 70% ethanol. Tissues were embedded in paraffin, and sections were hematoxylin and eosin stained. To quantify villus height and crypt depth, four villi and four crypts were measured from the distal and proximal parts of the perfused intestinal segments from each rat using the software Zeiss Zen 3.9, and the mean villus length and crypt depth were calculated.

### 2.9 Expression of nutrient transporters and brush border enzymes

Biopsies were kept at 4°C in RNAlater (Sigma Aldrich, cat no R0901) until homogenization in RLT buffer using a tissue lyzer and RNA extraction using RNeasy^®^ Mini kit (Qiagen, cat no. 74104). The amount of RNA obtained was quantified using NanoDrop™. cDNA synthesis from RNA was done using SuperScript III Reverse Transcriptase (Invitrogen, cat no. 12087539); 4 μL 5X First‐Strand Buffer, 1 μL random primers, 1 μL 0.1 M DTT and 10 μL of diluted RNA were added. Samples were heated at 70°C for 3 min. Following this, 1 μL 10 mM dNTP mix, 1 μL RNaseOUT™ Recombinant RNase Inhibitor (Invitrogen, cat no. 10777019), 1 μL of SuperScript™ III RT, and 2 μL of MilliQ water were added to each microcentrifuge tube. The samples were heated as follows: 25°C for 5 min, 50°C for 60 min, 70°C for 15 min and then cooled to 4°C. For quantitative RT‐PCR, reactions were set up using SYBR^®^ Green PCR Master Mix (Applied Biosystems, cat no. A25741) alongside the cDNA template, forward and backward primers and MilliQ water. The expression of genes was normalized to the GAPDH using the ΔΔCT method. The primers used for expression analysis are shown in Table S1.

### 2.10 Biochemical measurements

Glucose concentration from the venous effluent, which was collected continuously each minute, was measured during the perfusion every third minute by glucometer (Accu‐Chek^®^ Mobile; Roche Diagnostics A/S, Denmark). Free fatty acids were measured every third minute using the NEFA‐HR(2) assay (ref. no. 434‐91795; FUJIFILM Wako Chemicals, Germany). Total L‐Amino Acids were measured every third minute using an L‐Amino Acid assay kit (Abcam, Cambridge, United Kingdom, cat no. ab65347). Total GLP‐1 concentrations, representing the sum of GLP‐1(7–36)NH_2_, GLP‐1(9–36)NH_2_, and any fragments cleaved mid‐terminally, were measured in the venous effluent using a validated C‐terminal in‐house radioimmunoassay, codename 89390 (Orskov et al., 1994).

### 2.11 Individual amino acid measurements

Amino acids were measured using low‐resolution liquid chromatography coupled to mass spectrometry as described in the Appendix S1. Briefly, samples were extracted using a mixture of acetonitrile, methanol and water containing isotopically labeled standards. The extracts were incubated at −20°C for 30 min and then centrifuged. Clarified supernatants were transferred to autosampler vials, which were placed in an autosampler and injected for analysis using positive, single ion monitoring. Metabolites were quantified using a standard curve containing isotopically labeled standards at the same concentration as in the samples. The liquid chromatography gradient, single ion monitoring parameters, and mass spectrometer parameters are specified in Tables S5–S7, respectively.

### 2.12 Measurement of superior mesenteric artery blood flow

Superior mesenteric artery blood flow was measured in non‐fasted anesthetized rats using an ultrasonic flow probe (Transonic 1PRB) as described in Galsgaard et al. (2025). The GLP‐2R agonist and GIPR agonist were dissolved in 0.9% saline +1% BSA and given as an intravenous (i.v.) bolus injection (100 μL) in the jugular catheter used for the continuous saline infusion. As a negative control, an i.v. bolus injection of 100 μL 0.9% saline +1% BSA was given. To evaluate the effects of the bolus injections on blood flow, mean arterial blood pressure, and heart rate, recordings were made every second, and values were extracted every 10th second from 1 min before the bolus injection until 2 min after the bolus injection. The post‐injection recordings shown in the line graphs in the flow data figure are expressed as % changes from baseline, calculated as ((stimulation‐baseline)/baseline) × 100%. The stimulation is the actual value extracted every 10th second up to 2 min after the bolus injection. The baseline is the mean of the actual values extracted every 10th second for 1 min before the bolus injection. The bar graphs show the effect of the bolus injections calculated as follows: ((stimulation‐baseline)/baseline) × 100%. The baseline is the mean of the actual values extracted every 10th second 1 min before the bolus injection. The stimulation is the mean of the actual values extracted every 10th second up to 2 min after the bolus injection as also described in Galsgaard et al. (2025). All the individual experiments are shown in Figures S6 and S8.

### 2.13 Statistics

Values are presented as mean ± standard deviation (SD). Groups were compared using Brown‐Forsythe and Welch one‐way ANOVA with a post hoc Dunnett's correction for multiple testing, with the exception of the flow data which were analyzed by repeated measurement mixed‐effects analysis, corrected for multiple testing using the Tukey test. A *p* value of less than 0.05 was considered statistically significant. All statistical analyses were carried out using GraphPad Prism 10.2.0 (La Jolla, CA, USA).

## 3 RESULTS

### 3.1 The GLP‐2R agonist activates the rat GLP‐2R and the GIPR agonist activates the rat GIPR

**FIGURE 1:**
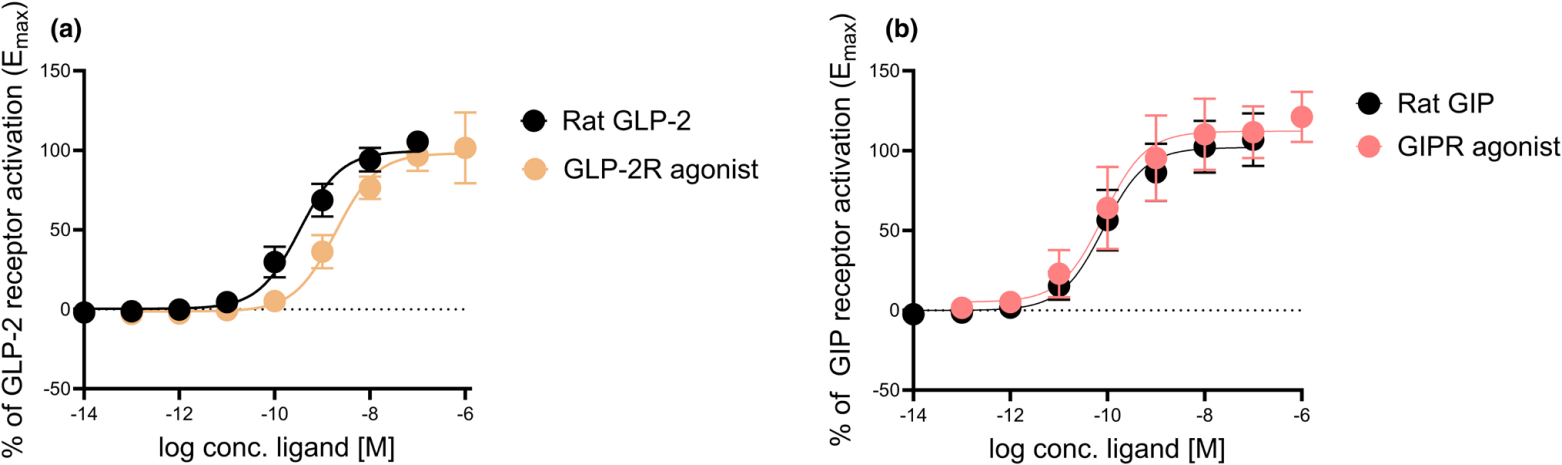
The GLP‐2R agonist activates the rat GLP‐2R and the GIPR agonist activates the rat GIPR. In vitro pharmacological characterization of (a) the GLP‐2R agonist on the rat GLP‐2R compared to rat GLP‐2 (*n* = 6 and 6, respectively, and *n* = 2 technical replicates for both) and (b) the GIPR agonist on the rat GIPR compared to rat GIP (*n* = 3 and 9 biological replicates, respectively and *n* = 2 technical replicates for both). (a) and (b) are dose–response curves and the read‐out is cAMP accumulation in transiently transfected COS‐7 cells.

We first characterized the activity of the GLP‐2R agonist and GIPR agonist compared to rat GLP‐2(1‐33) and rat GIP(1‐42) on cells transfected with the rat GLP‐2R and rat GIPR, respectively. The GLP‐2R agonist activated the rat GLP‐2R with a somewhat lower potency than that of rat GLP‐2 (LogEC_50_ = −8.7 ± 0.1 and LogEC_50_ = −9.5 ± 0.1, respectively) (Figure 1a), whereas the GIPR agonist activated the rat GIPR with a potency identical to that of rat GIP(1‐42) (LogEC_50_ = −10.1 ± 01 and LogEC_50_ = −10.1 ± 0.1, respectively) (Figure 1b). The potencies of the native rat hormones were previously published in Galsgaard et al. (2025).

### 3.2 Chronic treatment with the GLP‐2R agonist induces intestinal hypertrophy

**FIGURE 2:**
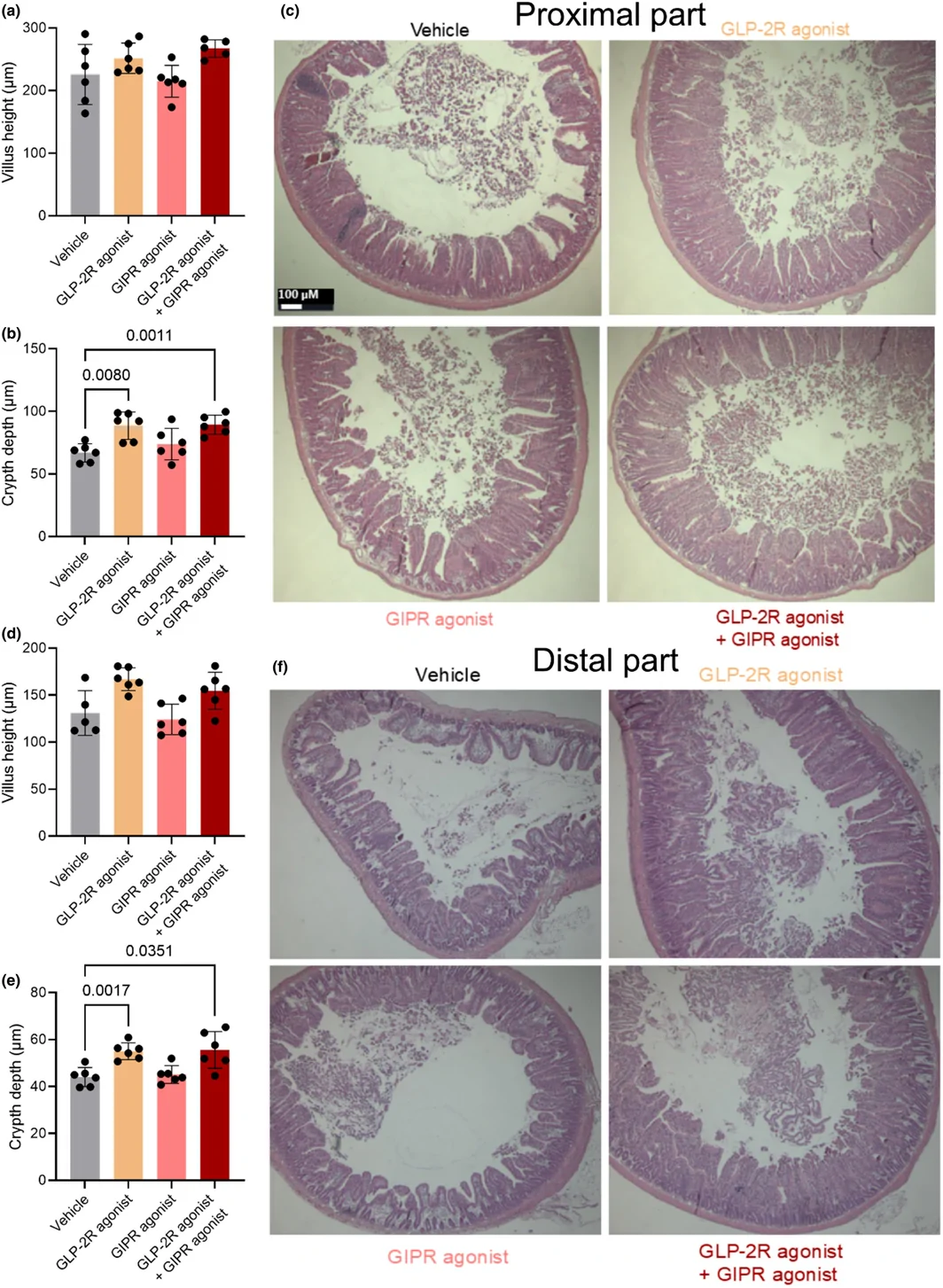
Chronic treatment with the GLP‐2R agonist induces intestinal hypertrophy. Villus heights in the (a) proximal and (d) distal part of the perfused intestinal segment in rats chronically (6 days) treated with vehicle (gray/black), GLP‐2R agonist (yellow), GIPR agonist (pink), or GLP‐2R agonist + GIPR agonist (red). Representative H&E staining's of the (c) proximal and (f) distal part of the perfused intestinal segment. Crypt depths in the (b) proximal and (e) distal part of the perfused intestinal segment. Data shown as means±SD, *n* = 5–6. *p* Value by Brown‐Forsythe and Welch one‐way ANOVA, all compared to vehicle.

Following chronic (6 days) treatment with the GLP‐2R agonist, GIPR agonist, or GLP‐2R agonist + GIPR agonist, the intestinal weight to length ratio was increased in rats treated with GLP‐2R agonist (0.13 ± 0.008 g/cm) and GLP‐2R agonist + GIPR agonist (0.13 ± 0.008 g/cm) when compared to vehicle treated rats (0.1 ± 0.006 g/cm) (*p* < 0.0001 and *p* = 0.0002, respectively). The weight to length ratio was similar in GIPR agonist (0.1 ± 0.01 g/cm) and vehicle treated rats (*p* = 0.2). The villus height was similar in rats treated with GLP‐2R agonist and GLP‐2R agonist + GIPR agonist when compared to vehicle both in the proximal and distal part of the perfused intestinal segment (*p* > 0.5 and *p* > 0.2, respectively) (Figure 2a,d). The crypt depth was significantly increased in rats treated with GLP‐2R agonist and GLP‐2R agonist + GIPR agonist when compared to vehicle both in the proximal and distal part of the perfused intestinal segments (*p* < 0.009 and *p* < 0.04, respectively) (Figure 2b,e). No effect was observed upon GIPR agonist treatment alone. Representative sections of the proximal and distal part of the perfused intestinal segments are shown in Figure 2c,f, respectively.

### 3.3 Chronic GLP‐2R agonist and GIPR agonist treatment stimulated glucose absorption in the perfused rat small intestine

**FIGURE 3:**
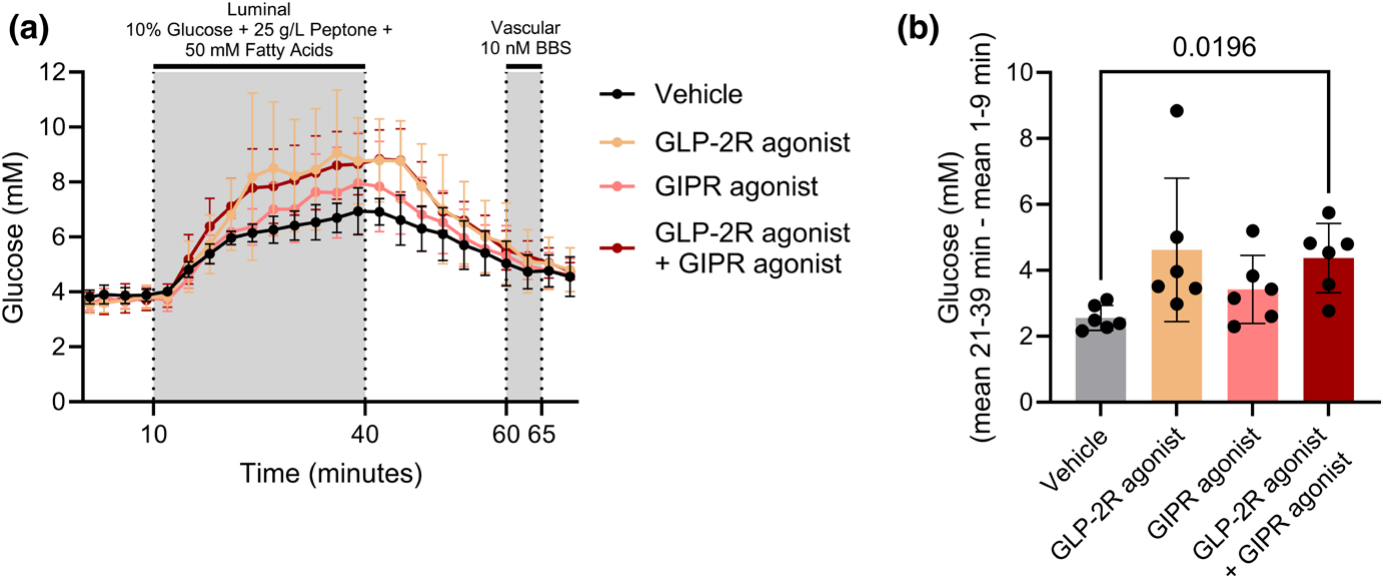
Glucose absorption after chronic treatment with GLP‐2R agonist + GIPR agonist in the isolated perfused rat proximal small intestine. (a and b) glucose concentrations in the effluent during perfusion of the proximal small intestine in rats chronically (six days) treated with vehicle (gray/black), GLP‐2R agonist (yellow), GIPR agonist (pink), or GLP‐2R agonist + GIPR agonist (red). Data shown as means±SD, *n* = 6. *p* Value by Brown‐Forsythe and Welch one‐way ANOVA, all compared to vehicle.

We measured blood glucose concentrations before the perfusion experiments to investigate whether this was affected by agonist treatments; there were no differences between the four groups: Vehicle 8.1 ± 0.9 mM, GLP‐2R agonist 7.5 ± 0.9 mM, GIPR agonist 7.7 ± 1.1 mM, and GLP‐2R agonist + GIPR agonist 7.4 ± 0.5 mM (*p* > 0.6). During the luminal nutrient perfusion of the proximal small intestine (Figure 3), glucose absorption increased in all groups. Glucose absorption was significantly higher in rats chronically treated with GLP‐2R agonist + GIPR agonist when compared to vehicle (*p* = 0.02). Glucose absorption was similar in vehicle and GIPR agonist treated rats (*p* = 0.3). This was also the case in GLP‐2R agonist treated rats; however, after exclusion of the highest value (that of 8.8 mM) in the GLP‐2R group, a *p* value of 0.047 was reached (Figure 3a,b). Chronic treatment with GLP‐2R agonist, GIPR agonist, and GLP‐2R agonist + GIPR agonist had no effect on the absorption of short‐ and medium‐chain fatty acids (*p* > 0.4) or amino acids (*p* > 0.1) (Figure S1). When adjusting the absorption rates to the flow and length of the perfused intestinal segments, similar results were obtained.

### 3.4 Subacute GLP‐2R agonist and GIPR agonist treatment did not affect glucose absorption in the perfused rat small intestine

**FIGURE 4:**
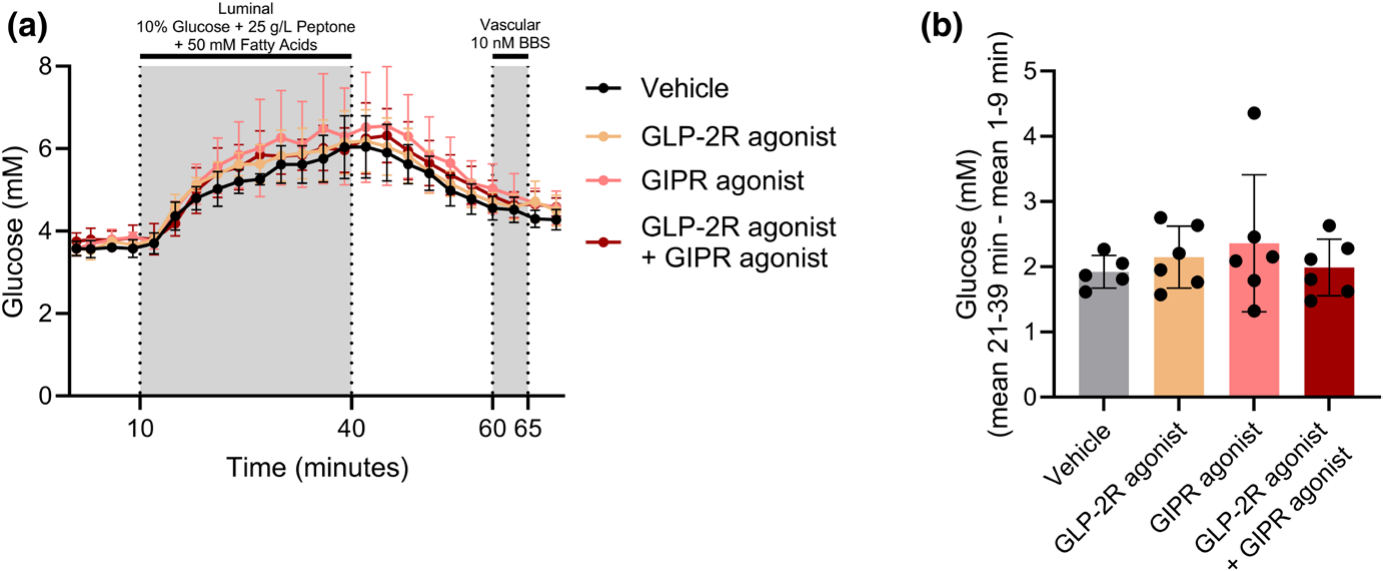
Subacute GLP‐2R agonist and GIPR agonist treatments do not affect glucose absorption in the perfused rat small intestine. (a and b) glucose concentrations during perfusion of the proximal small intestine in rats sub‐acutely (2 h) treated with vehicle (gray/black), GLP‐2R agonist (yellow), GIPR agonist (pink), or GLP‐2R agonist + GIPR agonist (red). Data shown as means±SD, *n* = 5–6. Nonsignificant *p* values were obtained by Brown‐Forsythe and Welch one‐way ANOVA, all compared to vehicle.

Subacute (2 h) pretreatment with GLP‐2R agonist, GIPR agonist, and GLP‐2R agonist + GIPR agonist had no effect on glucose (*p* > 0.6) (Figure 4a,b) or short‐ and medium‐chain fatty acid (*p* > 0.5) (Figure S2) absorption in the perfused rat proximal small intestine compared to vehicle. Also, when adjusting the absorption rates to the flow and length of the perfused intestinal segment, similar results were obtained.

### 3.5 Acute GLP‐2 and GIP treatment did not increase glucose absorption in the perfused rat small intestine

**FIGURE 5:**
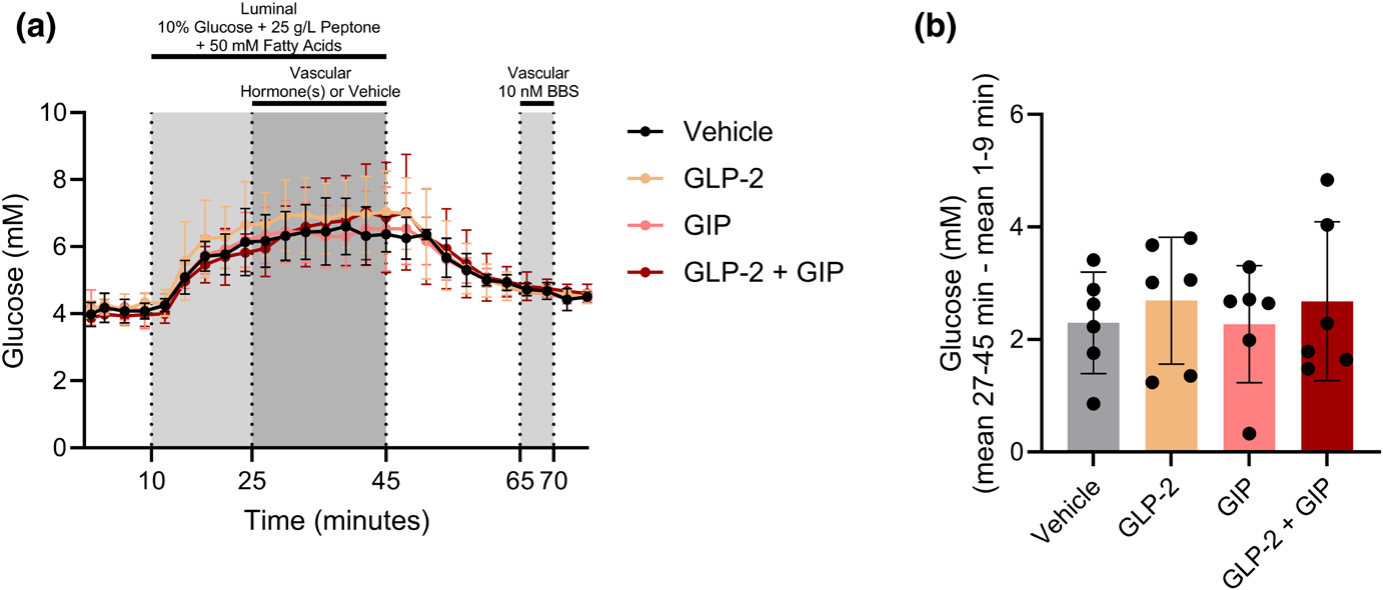
Acute GLP‐2 and GIP treatment does not increase glucose absorption in the perfused rat small intestine. (a and b) glucose concentrations during perfusion of the proximal small intestine in rats acutely (intravascularly during the perfusion) treated with vehicle (gray/black), native human GLP‐2 (yellow), native human GIP (pink), or GLP‐2 + GIP (red). GLP‐2, GIP, or GLP‐2 + GIP were infused intravascularly at a rate of 0.375 mL/min reaching a final concentration of 10 nM each. Data shown as means±SD, *n* = 6. Nonsignificant *p* values were obtained by Brown‐Forsythe and Welch one‐way ANOVA, all compared to vehicle.

Acute treatment (intra‐arterially during the perfusion) with native GLP‐2, GIP, and GLP‐2 + GIP had no effect on glucose (*p* > 0.8) (Figure 5a,b), short‐ and medium‐chain fatty acid (*p* > 0.7), or amino acid absorption (Figure S3) (*p* > 0.6). Once again, similar results were obtained when adjusting the absorption rates to the flow and length of the perfused intestinal segment.

### 3.6 Absorption of individual amino acids in the isolated perfused rat intestine

To further understand the absorption of amino acids in the isolated perfused intestine, the concentration of individual amino acids was measured in perfusion samples from each group. The data is shown in Figure S4 and the raw baseline and stimulation values are shown in Tables S2–S4.

### 3.7 Acute GIP and GLP‐2 + GIP treatment increased the expression of GLUT2 in the proximal small intestine

**FIGURE 6:**
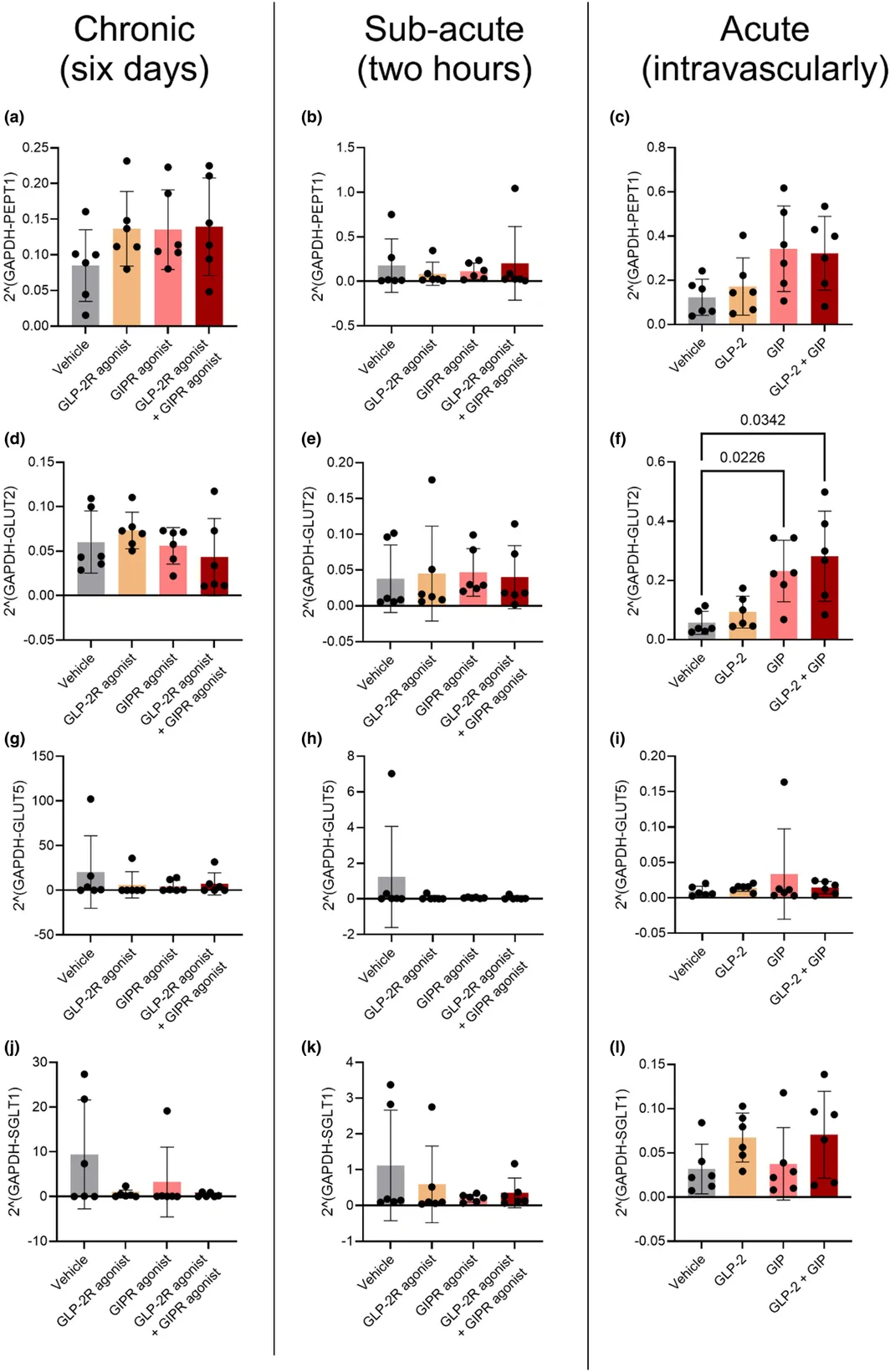
Expression of nutrient transporters following perfusion of the isolated proximal small intestine. Quantitative RT‐PCR of (a–c) PEPT1, (d–f) GLUT2, (g–i) GLUT5, (j–l) SGLT1 in rats treated (a, d, g, and j) chronically (6 days) and (b, e, h, and k) sub‐acutely (2 h) with vehicle, GLP‐2R agonist, GIPR agonist, GLP‐2R agonist + GIPR agonist and (c, f, i, and l) in rats treated acutely (intravascularly during the perfusion) with vehicle, GLP‐2, GIP, and GLP‐2 + GIP. Data shown as means±SD, *n* = 6. *p* Value by Brown‐Forsythe and Welch one‐way ANOVA, all compared to vehicle.

Chronic GLP‐2R agonist, GIPR agonist, and GLP‐2R agonist + GIPR agonist treatment did not change the expression of PEPT1, GLUT2, GLUT5, or SGLT1 (*p* > 0.3) (Figure 6a,d,g,j), neither did subacute GLP‐2R agonist, GIPR agonist, and GLP‐2R agonist + GIPR agonist treatment (*p* > 0.4) (Figure 6b,e,f,k). Acute GIP and GIP + GLP‐2 treatment surprisingly increased the expression of GLUT2 (*p* < 0.03 and *p* < 0.04, respectively) (Figure 6f), but not GLUT5, PEPT1, or SGLT1 (*p* > 0.09) (Figure 6c,i,l).

### 3.8 Chronic GLP‐2 and GIP treatment decreases nutrient‐stimulated GLP‐1 secretion in the perfused rat small intestine whereas acute treatment increases it

**FIGURE 7:**
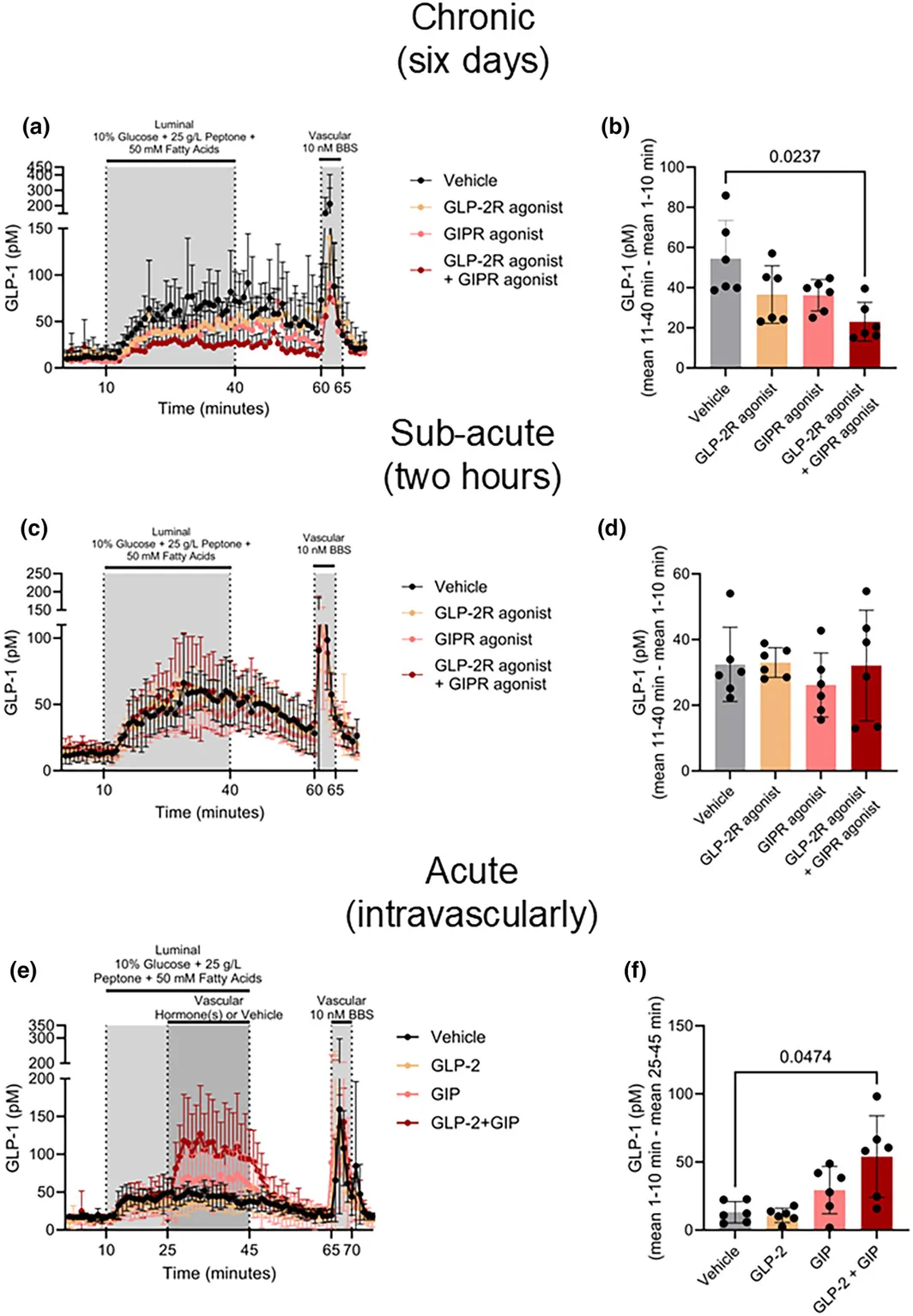
Chronic GLP‐2 and GIP treatment decreases nutrient‐stimulated GLP‐1 secretion in the perfused rat small intestine whereas acute treatment increases it. Nutrient stimulated GLP‐1 secretion in rats upon (a and b) chronic (6 days) treatment with vehicle (gray/black), GLP‐2R agonist (yellow), GIPR agonist (pink), or GLP‐2R agonist + GIPR agonist (red). (c and d) subacute (2 h) treatment with vehicle (gray/black), GLP‐2R agonist (yellow), GIPR agonist (pink), and GLP‐2R agonist + GIPR agonist (red), and (e and f) acute (intravascularly during the perfusion) treatment with vehicle (gray/black), GLP‐2 (yellow), GIP (pink), or GLP‐2 + GIP (red). Data shown as means±SD, *n* = 5–6. *p* Value by Brown‐Forsythe and Welch one‐way ANOVA, all compared to vehicle.

During the perfusion of the proximal small intestine, GLP‐1 secretion (stimulated by the luminal infusion of 10% glucose, 25 g/L peptone, and 50 mM short‐ and medium‐chain fatty acids) was approximately halved (fold change 0.4) in rats treated chronically with GLP‐2R agonist + GIPR agonist compared to vehicle (*p* < 0.03). No significant effect on GLP‐1 secretion was observed following chronic GLP‐2R agonist and GIPR agonist treatment alone (*p* = 0.3 and *p* = 0.2, respectively) (Figure 7a,b). Subacute (2 h) treatment with GLP‐2R agonist, GIPR agonist, and GLP‐2R agonist + GIPR agonist had no effect on GLP‐1 secretion (*p* > 0.6) (Figure 7c,d). Acute treatment with native GLP‐2 in combination with GIP increased GLP‐1 secretion by 4‐fold (*p* = 0.047), and GIP tended to increase secretion (*p* = 0.2) whereas GLP‐2 alone had no effect on GLP‐1 secretion (*p* > 0.1) (Figure 7e,f).

### 3.9 Nutrient absorption is dependent on the vascular perfusion flow rate in the isolated vascularly perfused rat proximal small intestine

**FIGURE 8:**
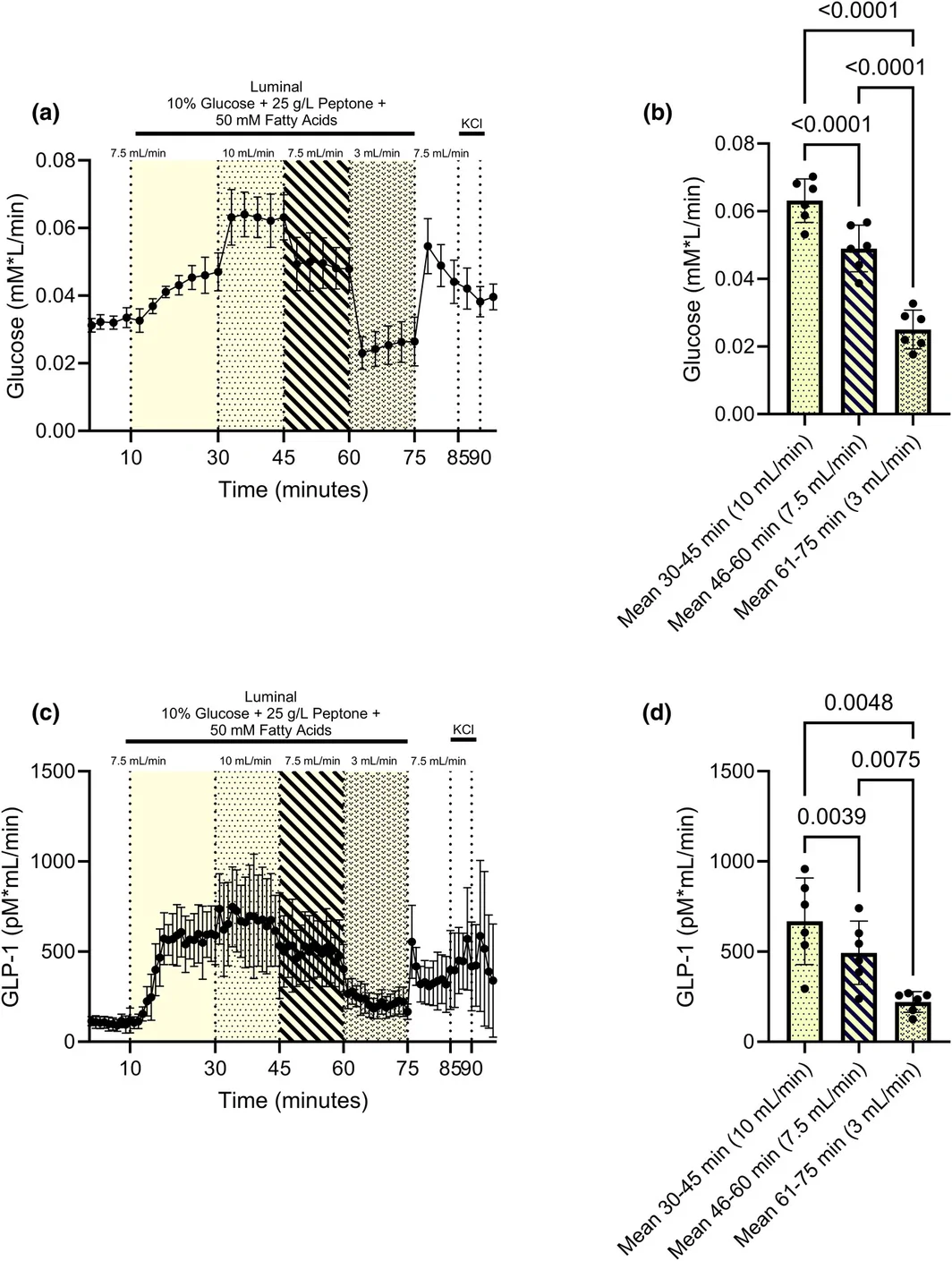
Nutrient absorption is dependent on the vascular perfusion flow rate in the isolated vascularly perfused rat proximal small intestine. (a and b) glucose and (c and d) GLP‐1 concentrations during perfusion of the proximal small intestine in rats at different vascular perfusion flow rates indicated in the figures. Data shown as means±SD, *n* = 5–6. *p* Value by Brown‐Forsythe and Welch repeated measurements one‐way ANOVA.

In the isolated perfused intestinal model, a catheter is inserted into the superior mesenteric artery and through this perfusion buffer (replacing blood) is infused. Thus, the stimulatory effect of GLP‐2 and GIP on superior mesenteric blood flow is abolished in this model and the perfusion buffer is infused at a constant rate (7.5 mL/min). This was also the case for the perfusion experiments described so far investigating the effects of the receptor agonists, GLP‐2, and GIP. To investigate if nutrient absorption is dependent on the flow through the superior mesenteric artery, we conducted a series of experiments in which the arterial perfusion flow rate (via the superior mesenteric artery) was increased from 7.5 mL/min to 10 mL/min and then decreased to 3 mL/min. The increased flow resulted in a 1.3‐fold increase in glucose output in the venous effluent (*p* < 0.0001), whereas the decrease in flow halved the glucose output (*p* < 0.0001) (Figure 8a,b). The output of short‐ and medium‐chain fatty acids and amino acids was also dependent on the flow rate as the output increased with the increased flow rate (*p* < 0.002 and *p* = 0.03, respectively) and decreased with the decrease in flow rate (*p* < 0.0004 and *p* = 0.09, respectively) (Figure S5). In line with increased glucose output, GLP‐1 secretion increased with the increased flow rate (*p* < 0.004) and decreased with the decrease in flow rate (*p* < 0.008) (Figure 8c,d).

### 3.10 The GIPR agonist, but not GLP‐2R agonist, increased superior mesenteric artery blood flow in anesthetized rats

**FIGURE 9:**
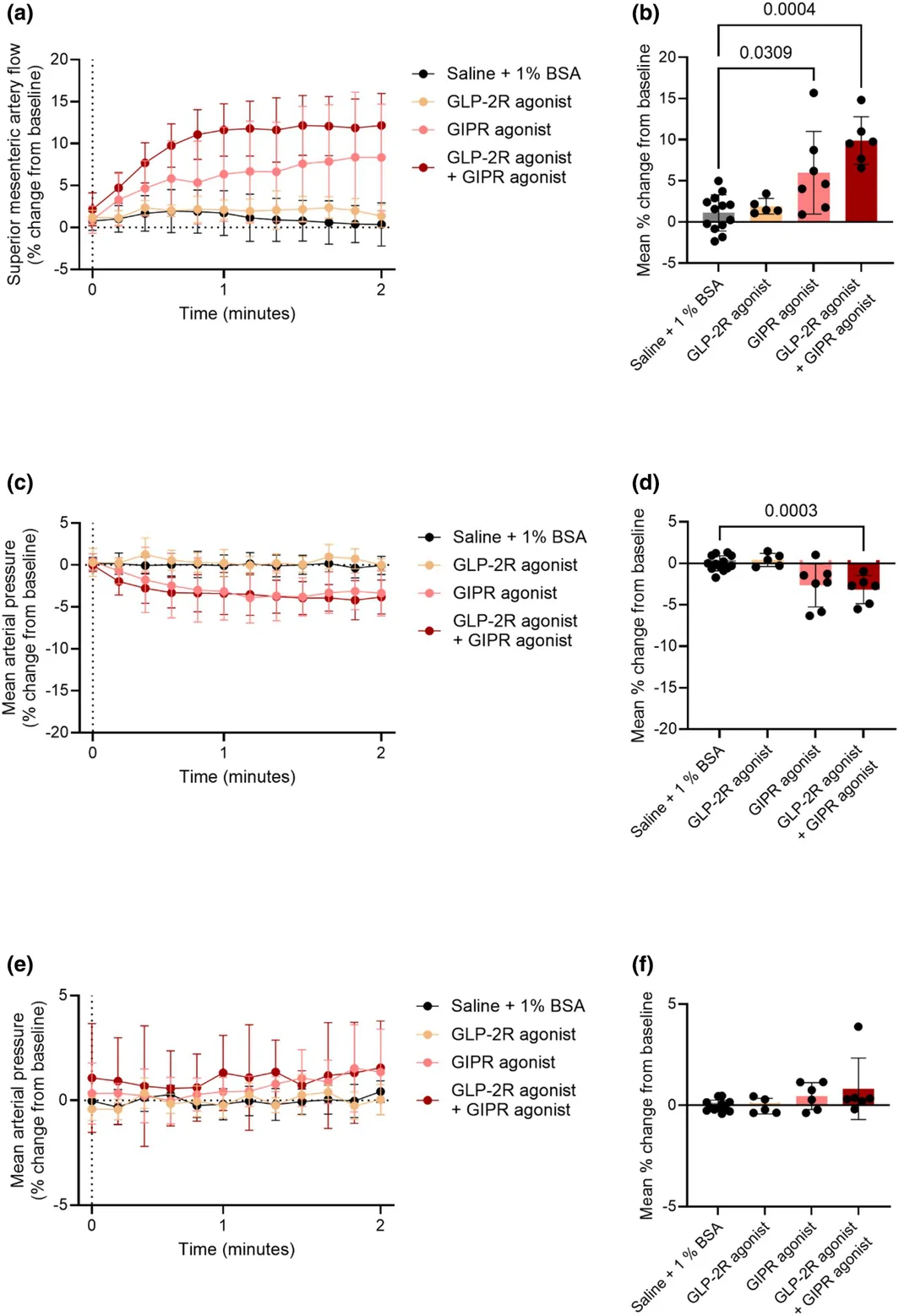
The GLP‐2R agonist and GIPR agonist may act synergistically to increase superior mesenteric artery blood flow. % change in (a and b) **s**uperior mesenteric artery blood flow, (c and d) mean arterial blood pressure, and (e and f) heart rate in response to an intravenous bolus injection of saline +1% BSA, 0.008 mg pr rat GLP‐2R agonist, 0.0096 mg pr rat GIPR agonist, and 0.004 mg per rat GLP‐2R agonist +0.0048 mg per rat GIPR agonist. Data shown as means±SD, *n* = 5–13. *p* Values by repeated measurement mixed‐effects analysis, all groups compared corrected for multiple testing using the Tukey test. Due to the experimental setup, the saline controls are identical to those published in Galsgaard et al. (2025) in Figure 4.

As both GLP‐2 and GIP are potent stimulators of superior mesenteric artery blood flow and this might influence nutrient uptake, we investigated whether the GLP‐2R agonist and GIPR agonist changed superior mesenteric artery blood flow in vivo. The GLP‐2R agonist did not increase superior mesenteric blood flow when compared to saline (1.9% ± 1.0% vs. 1.1% ± 2.2%) (*p* > 0.9), whereas the GIPR agonist increased superior mesenteric blood flow by 6.0 ± 5.0% (*p* < 0.04). When the doses of the two receptor agonists were halved and combined, the blood flow increased by 9.9 ± 2.8% (*p* < 0.0005), however, the difference was not significant when the effect of the combination was compared to that of the GLP‐2R agonist (*p* > 0.2) or GIPR agonist alone (*p* > 0.4) (Figure 9a,b). The GLP‐2R agonist did not affect blood pressure when compared to saline (*p* > 0.3), whereas the GIPR agonist tended to decrease blood pressure (by −2.7 ± 2.6%) when compared to saline (*p* < 0.08). GLP‐2R agonist + GIPR agonist decreased mean arterial blood pressure by −3.2 ± 1.7% (*p* < 0.0004) (Figure 9c,d). The heart rate was not affected by the receptor agonists (*p* > 0.3) (Figure 9e,f). These flow data were derived from an extended protocol investigating the effects of native GLP‐2 and GIP, and therefore the saline controls are the same saline controls as those for the experiment published in Galsgaard et al. (2025) in Figure 4.

When administered in a 50 times higher dose, the GLP‐2R agonist increased superior mesenteric artery blood flow by 8.7 ± 5.5%; however, this was not significant when compared to saline, most likely due to the low number of experiments (1.9 ± 4%) (*p* < 0.2). In a 50 times higher dose, the GIPR agonist increased superior mesenteric artery blood flow by 21.5 ± 7.4% (*p* < 0.03). In these doses, neither of the receptor agonists consistently affected mean arterial blood pressure (*p* > 0.6 and *p* > 0.8, respectively) or heart rate (*p* > 0.7 and *p* > 0.4, respectively) (Figures S7 and S8).

## 4 DISCUSSION

In this study, we investigated whether GLP‐2 and GIP alone or combined would enhance intestinal absorption of glucose, short‐ and medium‐chain fatty acids, and amino acids using the isolated perfused rat proximal small intestine model. Our hypothesis that GLP‐2 and GIP might have a synergistic effect on nutrient absorption was not supported by our studies that suggest that subacute and acute GLP‐2 and GIP treatment do not measurably affect glucose, short‐ and medium‐chain fatty acid, or amino acid absorption during perfusion with a constant arterial flow rate. Confirmatory to previous observations, chronic GLP‐2R agonist treatment increased intestinal size. Chronic GLP‐2R agonist increased glucose absorption and the effect became significant when combined with GIPR agonist treatment. Intestinal nutrient absorption increased with the vascular perfusion flow rate.

Glucose absorption was statistically increased in rats that were simultaneously treated chronically with both the GLP‐2R agonist and GIPR agonist and tended to be increased with GLP‐2R agonist treatment alone. As the chronic treatment with the GLP‐2R agonist markedly increased the weight‐to‐length ratio of the small intestine after 6 days of treatment, the increase in glucose absorption could simply be attributed to an increased surface area of the intestine; however, this was not reflected by an increased absorption of short‐ and medium‐chained fatty acids or amino acids, and thus a specific effect of chronic GLP‐2 treatment on glucose absorption cannot be excluded. We observed no effects of chronic or subacute GLP‐2R agonist, GIPR agonist, or GLP‐2R agonist + GIPR agonist treatment on nutrient transporter expression (PEPT1, SGLT1, GLUT2, and GLUT5), but surprisingly acute GIP and GLP‐2 + GIP increased the expression of GLUT2. These results were not supported by protein expression analysis, and it is therefore difficult to draw any firm conclusions.

GLP‐2 administration has in some studies been shown to stimulate glucose and fructose uptake, increase protein expression of SGLT1, GLUT2, and PEPT1, as well as increase the activity of SGLT1 (Au et al., 2002; Cheeseman, 1997; Hu et al., 2010; Ramsanahie et al., 2003). In contrast, a single study showed that GLP‐2 administration lowered intestinal SGLT1 and GLUT2 expression and did not affect absorption of oral or duodenal administered glucose or maltose. In that study, GLP‐2 treatment did induce small intestinal growth and increased activity of duodenal maltase, sucrase, lactase, glutamyl transpeptidase, and dipeptidyl‐peptidase IV (Brubaker et al., 1997). GLP‐2 administration has been reported to increase the absorption of amino acids in vivo, and GLP‐2R knockout mice showed reduced lysine transport (Brubaker et al., 1997; Lee et al., 2017). GIP administration has been reported to increase dipeptide and glucose transport, and the glucose effect was dependent on SGLT1 activity (Coon et al., 2013; Singh et al., 2008). GIP has also been reported to increase PEPT1 expression (Coon et al., 2015). This contrasts with our observations using the isolated perfused rat proximal small intestine, as we did not observe an effect of the GLP‐2R agonist, the GIPR agonist, or the combination on absorption of amino acids. Most of the models referenced above involve modifications of the intestinal mucosa and this may influence the results. We chose the isolated vascularly perfused preparation of the proximal rat small intestine for our studies, because this method allows absorption studies to be carried out in real time in intact and unmodified tissue, where vascular perfusion simultaneously allows effective draining of the absorbed nutrients preventing substrate inhibition of the transported nutrients. Indeed, the intestinal preparations effectively absorbed both glucose, amino acids, and short‐ and medium‐chain fatty acids, and the hormonal responses to the luminally administered nutrients and positive control at the end of the experiments illustrate the intact performance of the model. Unfortunately, absorption of long‐chain fatty acids cannot be studied very well in this model since the lymphatic drainage cannot be collected. Thus, long‐chain fatty acids were not included in the nutrient solution infused intra‐luminally as these are absorbed into the lymphatics rather than the portal vein, whereas short‐ and medium‐chain fatty acids should be passively absorbed into the portal vein, as also indicated by the results. GLP‐2 has been shown to have a mobilizing effect on intestinal lipid stores in both humans (Dash et al., 2014; Meier et al., 2006; Xiao et al., 2019), hamsters (Hsieh et al., 2009), and mice (Grande et al., 2022). Similarly, in rats, GIP has been shown to increase lymph flow rate and regulate post‐absorptive lipid release from the intestine and, thus, promote post‐absorptive lipid release (Wang et al., 2025). We did not observe an effect of the GLP‐2R agonist, the GIPR agonist, or GLP‐2 and GIP, on absorption of the selected medium‐ and short‐chain fatty acids.

Increased blood flow to the intestine may be an important mediator of nutrient absorption, as absorbed nutrients are cleared more rapidly from the subepithelial tissue when capillary flow increases. However, the absorptive effects of GLP‐2 on amino acids have been suggested to be independent of the GLP‐2 mediated increase in intestinal blood flow, as ex vivo studies (jejunal tissue preparations mounted in an Ussing chamber, eliminating intestinal blood flow as a factor) have shown absorptive effects of GLP‐2 (Lee et al., 2017). Further, in humans, the GLP‐2 induced mobilization of intestinal lipid stores was not affected by a ~50% reduction of blood flow to the intestine (Xiao et al., 2019). We found that a high dose of the GLP‐2R agonist did stimulate superior mesenteric artery blood flow in vivo whereas both doses of the GIPR agonist potently stimulated superior mesenteric blood flow. This was surprising as we previously found native GLP‐2 to induce a greater increase in superior mesenteric artery blood flow than native GIP (Galsgaard et al., 2025). This could suggest that the long‐acting GLP‐2R agonist might not be as potent inducer of superior mesenteric artery vasodilation as when administered in its native form. In our perfusion model, the perfusion buffer is normally infused at a constant rate. This allows for investigations of nutrient absorption independently of blood flow, where potential effects of expression and translocation of nutrient transporters would be evident. The perfusion experiments investigating the effects of the receptor agonists, GLP‐2, and GIP, were done using a constant arterial flow rate, thus the effects of the receptor agonists, GLP‐2, and GIP on blood flow are not translated in these experiments. However, as GLP‐2 and GIP are potent stimulators of superior mesenteric artery flow in rats (Galsgaard et al., 2025), we wanted to test whether nutrient absorption depended on the vascular perfusion flow rate. We found that when the arterial flow through the superior mesenteric artery was increased from 7.5 mL/min to 10 mL/min, the output of glucose, short‐ and medium‐chain free fatty acids, and amino acids increased in the venous effluent, likewise it was lower when the flow rate was lowered to 3 mL/min. This supports the hypothesis that GLP‐2 and GIP might increase nutrient absorption by increasing superior mesenteric blood flow, which is in line with the lack of effects of the subacute and acute hormone stimulation on nutrient absorption in our perfusion model. The observation also supports the view that intestinal absorption is dependent on blood flow, consistent with the observation that intestinal blood flow increases several fold after meal ingestion (Bremholm et al., 2009) as well as in response to ingestion of isolated nutrients like glucose (Rasmussen et al., 2025).

Our three different treatment regimens allowed us to investigate the effects of chronic, subacute, and acute GLP‐2 and GIP treatment on GLP‐1 secretion in response to nutrients. Chronic treatment with the GLP‐2R agonist in combination with GIPR agonist reduced nutrient‐stimulated GLP‐1 secretion, and the GLP‐2R and GIPR agonists alone also tended to decrease GLP‐1 secretion. No effects were observed upon sub‐acute treatment. Perhaps chronic GLP‐2 treatment induces negative feedback on the L‐cells, from which both GLP‐2 and GLP‐1 are secreted. Chronic administration of the GIPR agonist may induce GIPR desensitization, as described for GIPRs in the pancreas (Davies et al., 2025), resulting in the known stimulatory effect of GIP on GLP‐1 secretion (Herrmann‐Rinke et al., 1995) being reduced. In the acute studies, a stimulatory effect of GIP, especially in combination with GLP‐2, was observed on GLP‐1 nutrient‐stimulated secretion.

A limitation of our study is that native human GLP‐2 and GIP were used to study the acute effects of the native hormones. This may have resulted in an underestimation of the effect of GIP, as human GIP is a comparatively weak agonist on the rat GIPR with an E_max_ of 75% and no differences in binding affinities (Sparre‐Ulrich et al., 2016), whereas human GLP‐2 has similar activation profiles on the rat and human GLP‐2R (Gadgaard et al., 2023). Similarly, the GIPR agonist activates the rat GIPR with a lower potency than rat GIP. Additionally, we only measured absorption of nutrients in a relatively short‐time interval (≤30 min) which might not have been sufficient to observe potential effects, and we only tested absorption of one concentration of the nutrients. Furthermore, the GLP‐2 and GIP concentrations infused intravascularly may have been supraphysiological, but the 10 nM concentration approaches C_max_ of the receptor binding relationships and was therefore chosen not to miss potential effects. The luminal infused glucose concentration may also be supraphysiological; however, at this rate glucose absorption is not saturated.

Our studies confirm the effects of GLP‐2 on intestinal growth and the effects of GIP on intestinal blood flow. Additionally, our studies provide important insights regarding the effects of GLP‐2 and GIP on intestinal parameters, suggesting that regulation of intestinal blood flow is an important mechanism influencing nutrient uptake.

## AUTHOR CONTRIBUTIONS

**Katrine D. Galsgaard:** Conceptualization; data curation; formal analysis; funding acquisition. **Tyler A. Cookson:** Conceptualization; data curation; formal analysis. **Ida M. Modvig:** Data curation. **Valentina Abba:** Data curation. **Samuel A. J. Trammell:** Data curation; formal analysis. **Mette M. Rosenkilde:** Data curation; formal analysis. **Lærke S. Gasbjerg:** Funding acquisition. **Charlotte M. Sørensen:** Funding acquisition; supervision. **Bolette Hartmann:** Conceptualization; funding acquisition; project administration; supervision. **Jens J. Holst:** Conceptualization; funding acquisition; project administration; supervision.

## FUNDING INFORMATION

K.D.G. is supported by the BRIDGE‐Translational Excellence Programme (bridge.ku.dk) at Faculty of Health and Medical Sciences, University of Copenhagen, Copenhagen, funded by the Novo Nordisk Foundation. Grant agreement no. NNF20SA0064340. J.J.H. is supported by The Independent Research Fund Denmark (DFF) (DFF 3101‐00127B) and the Novo Nordisk Foundation (NNF) Center for Basic Metabolic Research University of Copenhagen (NNF Application Number: 13563). Novo Nordisk Foundation Center for Basic Metabolic Research is an independent Research Center, based at the University of Copenhagen, Denmark and partially funded by an unconditional donation from the Novo Nordisk Foundation (Grant number NNF18CC0034900 and NNF23SA0084103).

## CONFLICT OF INTEREST STATEMENT

M.M.R., L.S.G., and J.J.H. are minority shareholders of Antag Therapeutics. M.M.R. and J.J.H. are also involved in advisory board activity. L.S.G. is involved in advisory board activity and has received speaker honorarium from Novo Nordisk. J.J.H. is co‐founder and member of the advisory board of Bainan Biotech. M.M.R. is co‐founder of Bainan Biotech. B.H. is co‐founder of Bainan Biotech.

## ETHICS STATEMENT

All animal studies were approved by the Danish Animal Experiments Inspectorate (2020‐15‐0201‐00547 and 2023‐15‐0201‐01408) and the local ethical committee (Department of Experimental Medicine).

## Supporting information


Appendix S1.

## Data Availability

All data reported in this paper will be shared by the lead contact upon request.
