# Supplementary material for: Chronic, but not acute, treatment with GLP‐2 and GIP in combination increases glucose absorption in the isolated vascularly perfused rat intestine
Source: Physiol Rep. 2026 Aug 25;14(16):e71069. doi: 10.14814/phy2.71069 (PMC13507514; doi:10.14814/phy2.71069)
Supplement: Supplementary file 1 [file PHY2-14-e71069-s001.docx]

# **Supplementary materials**

**Supplementary Figure 1. Nutrient absorption after chronic treatment with GLP-2R agonist + GIPR agonist in the isolated perfused rat proximal small intestine.** *A* and *B*) free fatty acid (NEFA), *C* and *D*) amino acid concentrations in the effluent during perfusion of the proximal small intestine in rats chronically (six days) treated with vehicle (grey/black), GLP-2R agonist (yellow), GIPR agonist (pink), or GLP-2R agonist + GIPR agonist (red). Data shown as means±SD, n=6. P-value by Brown-Forsythe and Welch one-way ANOVA, all compared to vehicle, *P<0.05.

**Supplementary Figure 2. Sub-acute GLP-2R agonist and GIPR agonist treatments do not affect free fatty acid absorption in the perfused rat small intestine.** *A* and *B*) free fatty acid (NEFA), concentrations during perfusion of the proximal small intestine in rats sub-acutely (two hours) treated with vehicle (grey/black), GLP-2R agonist (yellow), GIPR agonist (pink), or GLP-2R agonist + GIPR agonist (red). Data shown as means±SD, n=5-6. Nonsignificant P-values were obtained by Brown-Forsythe and Welch one-way ANOVA, all compared to vehicle.

**Supplementary Figure 3. Acute GLP-2 and GIP treatment does not increase nutrient absorption in the perfused rat small intestine.** *A* and *B*) free fatty acid (NEFA), *C* and *D*) amino acid concentrations during perfusion of the proximal small intestine in rats acutely (intravascularly during the perfusion) treated with vehicle (grey/black), native human GLP-2 (yellow), native human GIP (pink), or GLP-2 + GIP (red). GLP-2, GIP, or GLP-2 + GIP were infused intravascularly at a rate of 0.375 mL/min reaching a final concentration of 10 nM each. Data shown as means±SD, n=6. Nonsignificant P-values were obtained by Brown-Forsythe and Welch one-way ANOVA, all compared to vehicle.

**Supplementary Figure 4. Absorption of individual amino acids in the isolated perfused rat intestine.** The uptake of individual amino acids during perfusion of the proximal small intestine in rats upon *A*) chronic (six days), *B*) sub-acute (two hours) treatment with vehicle, GLP-2R agonist, GIPR agonist, or GLP2R agonist + GIPR agonist, and *C*) acutely (intravascularly during the perfusion) treated with vehicle, GLP-2, GIP, or GLP-2 + GIP. Perfusion samples from each group were pooled so that one baseline sample (the 5 min timepoint) and one stimulation sample (the 40 min timepoint) were analyzed for each group. To calculate the uptake of the individual amino acids the baseline amino acid value was subtracted from the amino acid value during stimulation.

**Supplementary Figure 5. Nutrient absorption is dependent on the vascular perfusion flow rate in the isolated vascularly perfused rat proximal small intestine.** *A* and *B*) free fatty acid (NEFA) and *C* and *D*), amino acid concentrations during perfusion of the proximal small intestine in rats at different vascular perfusion flow rates indicated in the figures. Data shown as means±SD, n=5-6. P-value by Brown-Forsythe and Welch repeated measurements one-way ANOVA.

**Supplementary Figure 6. The GLP-2R agonist and the GIPR agonist may act synergistically to increase superior mesenteric artery blood flow.** (*A*-*D*) Superior mesenteric artery blood flow, (*E*-*H*) mean arterial blood pressure, and (*I*-*L*) heart rate in response to an intravenous bolus injection of (*A, E, I*) 0.008 mg per rat GLP-2R agonist (*B, F, J*) 0.0096 mg per rat GIPR agonist, (*C, G, K*) 0.004 mg per rat GLP-2R agonist + 0.0048 mg per rat GIPR agonist, and (*D, H, L*) saline +1% BSA. Due to the experimental setup the saline controls are the same as those published in (39) Fig. 4.

**Supplementary Figure 7. The GIPR agonist increased superior mesenteric artery blood flow.** % change from baseline in (*A* and *B*) superior mesenteric artery blood flow, (*C* and *D*) mean arterial blood pressure, and (*E* and *F*) heart rate in response to an intravenous bolus injection of 0.4 mg per rat of GLP-2R agonist, 0.48 mg per rat of GIPR agonist, and saline. Data shown as mean±SD, n=4. P-values by repeated measurements one-way ANOVA, all compared to saline, corrected for multiple testing using the Dunnett test**.**

**Supplementary Figure 8. The GIPR agonist increases superior mesenteric artery blood flow.** (*A*-*C*) Superior mesenteric artery blood flow, (*E*-*F*) mean arterial blood pressure, and (*G*-*I*) heart rate in response to an intravenous bolus injection of (*A, D, G*) 0.4 mg of the GLP-2R agonist, (*B, E, H*) 0.48 mg of the GIPR agonist, and (*C, F, I*) saline.

| **Nutrient transporter** | **Primer set** |
| --- | --- |
| PEPT1 | Forward primer  CTCGGGGTTGCTTTGGCTAC  Reverse primer  GTGGAGAGGTCATCATCCCA |
| GLUT 2 | Forward primer GGGCAATCAGGATTACTGGCA  Reverse primer  TTCTGCTCAGTCGACGCCTCT |
| GLUT5 | Forward primer  TGCAGAGCAACGATGGAGAAA  Reverse primer  ACAGCAGCGTCAGGGTGAAG |
| SGLT1 | Forward primer  GACTGGGTTTGCTTTCCGAGA  Reverse primer  TGTTGGGTAGGCGATGTTGG |
| GAPDH | Forward primer CAACTCCCTCAAGATTGTCAGCAA  Reverse primer  GGCATGGACTGTGGTCATGA |

**Supplementary table 1. Primers used for nutrient transporter expression analysis.**

| **Amino acid concentrations in µmol/L** | **Vehicle** | **GLP-2R agonist** | **GIPR agonist** | **GLP-2R agonist +GIPR agonist** | **Vehicle** | **GLP-2R agonist** | **GIPR agonist** | **GLP-2R agonist +GIPR agonist** |
| --- | --- | --- | --- | --- | --- | --- | --- | --- |
|  | **Baseline** | | | | **Stimulation** | | | |
| **L-Alanine** | 100,51 | 181,66 | 104,14 | 153,01 | 160,27 | 212,74 | 162,07 | 214,31 |
| **L-Arginine** | 6,77 | 16,05 | 6,1 | 10,04 | 45,89 | 54,85 | 48,82 | 56,77 |
| **L-Asparagine** | 4,72 | 11,84 | 3,81 | 7,41 | 5,66 | 9,24 | 6,19 | 8,95 |
| **L-Aspartic acid** | 15,75 | 24,1 | 13,03 | 33,96 | 11,18 | 28,53 | 21,19 | 30,16 |
| **L-Glutamine** | 15,87 | 13,96 | 7,72 | 10,75 | 8,29 | 11,81 | 7,38 | 12,27 |
| **L-Histidine** | 3,66 | 6,91 | 3,01 | 4,77 | 4,13 | 5,92 | 4,32 | 6,1 |
| **L-Isoleucine** | 6,6 | 16,07 | 5,62 | 10,13 | 17,76 | 18,82 | 18,75 | 20,62 |
| **L-Leucine** | 12,4 | 29,26 | 11,73 | 18,57 | 30,56 | 35,11 | 32,59 | 35,41 |
| **L-Lysine** | 10,59 | 25,29 | 10,23 | 16,95 | 37,98 | 44,39 | 30,06 | 45,91 |
| **L-Methionine** | 2,88 | 7,52 | 2,65 | 4,55 | 3,24 | 4,65 | 3,8 | 4,51 |
| **L-Phenylalanine** | 5,27 | 12,62 | 5 | 7,97 | 18,03 | 22,25 | 18,85 | 22,86 |
| **L-Proline** | 11,77 | 21,1 | 10,21 | 14,47 | 57,74 | 60,47 | 53,12 | 66,48 |
| **L-Serine** | 9,43 | 15,09 | 9,29 | 12,38 | 21,84 | 24,83 | 15,54 | 32,3 |
| **L-Tryptophan** | 1,1 | 3,14 | NA | 1,9 | NA | 1,56 | 1,2 | 1,6 |
| **L-Tyrosine** | 4,02 | 9,76 | 3,35 | 6,48 | 6,7 | 8,84 | 7,64 | 9,74 |
| **L-Valine** | 9,84 | 21,54 | 8,55 | 14,31 | 27,88 | 31,97 | 28,1 | 34,58 |

**Supplementary table 2. Individual amino acid concentrations during perfusion of the isolated rat small intestine following chronic treatment (six days) with vehicle, a GLP-2R agonist, GIPR agonist, and GLP-2R agonist and GIPR agonist.** Perfusion samples from each group were pooled so that one baseline sample (the 5 min timepoint) and one stimulation sample (the 40 min timepoint) for each group were analyzed for concentrations of the individual amino acids using Liquid chromatography–mass spectrometry, NA denotes when concentrations were under the detection limit.

| **Amino acid concentrations in µmol/L** | **Vehicle** | **GLP-2R agonist** | **GIPR agonist** | **GLP-2R agonist +GIPR agonist** | **Vehicle** | **GLP-2R agonist** | **GIPR agonist** | **GLP-2R agonist +GIPR agonist** |
| --- | --- | --- | --- | --- | --- | --- | --- | --- |
|  | **Baseline** | | | | **Stimulation** | | | |
| **L-Alanine** | 86,75 | 91,68 | 87,73 | 103,27 | 148,55 | 139,52 | 141,68 | 140,45 |
| **L-Arginine** | 5,08 | 3,68 | 5,5 | 5,53 | 49,63 | 43,22 | 42,6 | 44,27 |
| **L-Asparagine** | 3,37 | 2,42 | 5,16 | 4,25 | 6,07 | 5,89 | 5,35 | 7,75 |
| **L-Aspartic acid** | NA | 13,26 | 10,02 | 13,56 | 13,77 | 13,31 | 11,18 | 13,11 |
| **L-Glutamine** | 6,05 | 7,06 | 6,59 | 7,23 | 8,08 | 8,29 | 7,23 | 8,23 |
| **L-Histidine** | 2,68 | 1,86 | 2,87 | 2,89 | 4,81 | 4,02 | 4,41 | 4,58 |
| **L-Isoleucine** | 4,01 | 3,51 | 6,25 | 5,22 | 17,53 | 14,12 | 15,52 | 16,75 |
| **L-Leucine** | 8,66 | 6,46 | 9,84 | 9,95 | 30,29 | 27,39 | 28,04 | 29,3 |
| **L-Lysine** | 8,09 | 6,9 | 8,68 | 9,39 | 40,93 | 28,67 | 27,71 | 28,32 |
| **L-Methionine** | 2,19 | 1,66 | 2,35 | 2,51 | 3,47 | 3,08 | 3,16 | 3,04 |
| **L-Phenylalanine** | 3,61 | 3,18 | 4,32 | 4,41 | 18,42 | 16,23 | 16,17 | 17,31 |
| **L-Proline** | 9,71 | 8,08 | 10,9 | 10,6 | 47,5 | 47,48 | 49,23 | 52,05 |
| **L-Serine** | 12,89 | 6,09 | 12,45 | 9,08 | 21,6 | 24,13 | 15,2 | 27,53 |
| **L-Tryptophan** | NA | NA | 1,08 | NA | 1,19 | NA | NA | 1,08 |
| **L-Tyrosine** | 3,18 | NA | 3,59 | 3,36 | 6,99 | 6,41 | 6,2 | 6,11 |
| **L-Valine** | 6,69 | 5,45 | 7,97 | 7,4 | 26,11 | 24,06 | 24,69 | 26,37 |

**Supplementary table 3. Individual amino acid concentrations during perfusion of the isolated rat small intestine following sub-acute treatment (2 hours) with vehicle, a GLP-2R agonist, GIPR agonist, and GLP-2R agonist and GIPR agonist.** Perfusion samples from each group were pooled so that one baseline sample (the 5 min timepoint) and one stimulation sample (the 40 min timepoint) for each group were analyzed for concentrations of the individual amino acids using Liquid chromatography–mass spectrometry, NA denotes when concentrations were under the detection limit.

| **Amino acid concentrations in µmol/L** | **Vehicle** | **GLP-2** | **GIP** | **GLP-2 + GIP** | **Vehicle** | **GLP-2** | **GIP** | **GLP-2 + GIP** |
| --- | --- | --- | --- | --- | --- | --- | --- | --- |
|  | **Baseline** | | | | **Stimulation** | | | |
| **L-Alanine** | 82,82 | 64,07 | 75,94 | 69,82 | 106,79 | 83,04 | 102,25 | 117,22 |
| **L-Arginine** | 4,1 | 3,5 | 3,43 | 3,86 | 27,47 | 26,11 | 25,8 | 34,24 |
| **L-Asparagine** | 3,59 | 3,18 | 2,08 | 2,18 | 4,43 | 2,46 | 4,17 | 5,16 |
| **L-Aspartic acid** | 11,37 | 11,03 | 10,11 | NA | NA | 10,2 | NA | NA |
| **L-Glutamine** | 5,57 | 5,62 | 5,04 | 4,42 | 5,02 | 4,78 | 4,98 | 5,51 |
| **L-Histidine** | 1,93 | 1,81 | 1,91 | 1,7 | 2,81 | 3,26 | 2,56 | 3,79 |
| **L-Isoleucine** | 4,61 | 3,82 | 3,32 | 3,39 | 11,1 | 8,66 | 9,85 | 14,14 |
| **L-Leucine** | 7,91 | 7,7 | 6,18 | 6,56 | 19,7 | 17,03 | 17,92 | 23,17 |
| **L-Lysine** | 5,75 | 5,76 | 7,57 | 6,51 | 18,82 | 15,63 | 17,26 | 23,14 |
| **L-Methionine** | 1,92 | 1,66 | 1,4 | 1,57 | 2,43 | 2,23 | 2,36 | 2,92 |
| **L-Phenylalanine** | 3,27 | 3,26 | 2,7 | 3,09 | 12 | 9,53 | 10,3 | 13,25 |
| **L-Proline** | 7,88 | 7,25 | 7,16 | 6,69 | 30,8 | 23,91 | 29,01 | 40,41 |
| **L-Serine** | 8,61 | 8,69 | NA | 5,53 | 14,8 | 14,29 | 14,94 | 15,26 |
| **L-Tryptophan** | NA | NA | NA | NA | NA | NA | NA | NA |
| **L-Tyrosine** | NA | NA | NA | NA | 4,82 | 3,79 | 4,31 | 5,01 |
| **L-Valine** | 5,84 | 5,41 | 5,18 | 4,52 | 16,41 | 15,34 | 15,43 | 20,05 |

**Supplementary table 4. Individual amino acid concentrations during perfusion of the isolated rat small intestine following acute treatment (intravascularly during the perfusion) with vehicle, GLP-2, GIP, and GLP-2 + GIP.** Perfusion samples from each group were pooled so that one baseline sample (the 5 min timepoint) and one stimulation sample (the 40 min timepoint) for each group were analyzed for concentrations of the individual amino acids using Liquid chromatography–mass spectrometry, NA denotes when concentrations were under the detection limit.

## *Individual amino acid measurements, detailed method description*

For the measurement of individual amino acids, perfusion samples from each group were pooled so that one baseline (5 min timepoint) and one stimulation sample (40 min timepoint) for each group were analyzed. 5 µL of perfusate was added to 95 µL of LC/MS grade acetonitrile:LC/MS grade methanol/filtered, deionized water (2:2:1) containing 375 pmol of amino acid internal standard (mixture from Cambridge Isotopes, Cat# MSK-A2-1.2; [^13^C_5_]-L-glutamine from Cambridge Isotopes, Cat# CLM-1822-H; [d_5_]-L-tryptophan from Cambridge Isotopes, Cat# DLM-1092; [^13^C_4_]-L-asparagine, Cat# CLM-8699-H). The samples were briefly vortexed at top speed for approximately one second and then placed in a freezer at -20°C for 30 min. Blanks were performed in triplicate by replacing the sample with water. Human plasma from Biowest (Cat# S4180-100) was also extracted in triplicate. After 30 min, the tubes were centrifuged for 10 min at 20,817×g with the temperature held at 20˚C. 60 µL of supernatant was transferred into an autosampler vials (Phenomenex Verex vials, Cat# AR0-3920-13). Equal volumes from all samples were combined in a vial and vortexed (as above) to compose the quality control pool sample. Quality control standards (0.6, 4 and 20 µM) and a standard curve (0.05, 0.15, 0.5, 1.5, 5, 15, 18.75, 20, 75, and 100 µM) were composed from L-amino acids (amino acid mixture from Merck Cat# AAS18-5ML, L-asparagine from Merck Cat# A8381, L-glutamine from Merck Cat# G5792 and L-tryptophan from Merck Cat# T8941) in the presence of the same concentration of internal standard as in samples. The quality control standards were performed in triplicate at each concentration level. The quality control standards and standard curve were composed in the same vials as samples, which were capped with the same caps as samples (Phenomenex Verex caps Cat# AR0-8952-13-M). All samples, quality controls, and standards were placed in a pre-chilled (6°C) autosampler and injected (2 µL) for LC/MS analysis. Samples were injected in a random order, and the injector was washed between injections using a mixture of 2-propnaol:methanol:acetonitrile:water (1:1:1:1). Samples were injected into an Agilent 1290 Infinity II UPLC system coupled to an Agilent LC/MSD-XT single quadrupole mass spectrometer. Amino acids were separated over a heated (35°C) Imtakt Intrada amino acid column (2.1 x 100 mm) with a gradient (table 1) between mobile phase A) 90 mM ammonium formate (Merck, Cat # 70221) and 666 µM oxalic acid (Merck, Cat # 658537) in LC/MS grade water (Fisher Scientific, Cat # 10777404) and mobile phase B) 0.3% (v/v) formic acid (VWR, Cat # 84865.260) in LC/MS grade acetonitrile (Fisher Scientific, Cat # 10001334)/water (Fisher Scientific, Cat # 10777404) (95/5% (v/v)). Amino acids and their heavy-labeled analogs were ionized using an Agilent Jet Stream ion source and detected in positive, single ion monitoring mode at mass-to-charge ratios and parameters described in Supplemental table 6. Other mass spectrometer parameters are detailed in Supplemental table 7. Peaks were detected based on retention time and mass-to-charge ratio and integrated using Agilent OpenLab CDS software. The pmol amounts were determined using an internally controlled standard curve and converted to micromolar by multiplying by the suspension volume (100 µL) and dividing by the injection volume (2 µL) and the volume of perfusate extracted (5 µL). The relative standard deviation for the human plasma extracts (Biowest, Cat# S4180-100) was below 15%. The QC standards differed from the theoretical concentration by less than 15%. Overall, reproducibility and accuracy were within the tolerance of 15% deviation.

| Time (min) | %B | Flow Rate (mL/min) |
| --- | --- | --- |
| 0 | 92 | 0.45 |
| 3 | 88 | 0.45 |
| 6.4 | 70 | 0.45 |
| 6.5 | 0 | 0.45 |
| 12 | 0 | 0.45 |
| 12.1 | 92 | 0.45 |
| 13.5 | 92 | 0.45 |

**Supplementary table 5. Liquid chromatography gradient.**

| Compound/Segment name | Mass (m/z) | Scan/Dwell time (ms) | Detector Gain Factor | Frag (V) |
| --- | --- | --- | --- | --- |
| [2H5]-D-Tryptophan | 210.1 | 11 | 1 | 100 |
| [13C3, 15N]-L-Alanine | 94.1 | 11 | 1 | 100 |
| [13C3, 15N]-L-Serine | 110.1 | 11 | 1 | 100 |
| [13C4, 15N]-L-Aspartic Acid | 139.1 | 11 | 1 | 100 |
| [13C4, 15N]-L-Threonine | 125.1 | 11 | 1 | 100 |
| [13C4]-L-Asparagine | 137.1 | 11 | 1 | 100 |
| [13C5, 15N]-L-Glutamic Acid | 154.1 | 11 | 1 | 100 |
| [13C5, 15N]-L-Methionine | 156.1 | 11 | 1 | 100 |
| [13C5, 15N]-L-Proline | 122.1 | 11 | 1 | 100 |
| [13C5, 15N]-L-Valine | 124.1 | 11 | 1 | 100 |
| [13C5]-L-Glutamine | 152.1 | 11 | 1 | 100 |
| [13C6, 15N]-L-(Iso)Leucine | 139.1 | 11 | 1 | 100 |
| [13C6, 15N2]-L-Cystine | 249 | 11 | 1 | 100 |
| [13C6, 15N2]-L-Lysine | 155.1 | 11 | 1 | 100 |
| [13C6, 15N3]-L-Histidine | 165.1 | 11 | 1 | 100 |
| [13C6, 15N4]-L-Arginine | 185.1 | 11 | 1 | 100 |
| [13C6,15N2]-L-Cysteine | 126 | 11 | 1 | 100 |
| [13C9, 15N]-L-Phenylalanine | 176.1 | 11 | 1 | 100 |
| [13C9, 15N]-L-Tyrosine | 192.1 | 11 | 1 | 100 |
| L-(Iso)Leucine | 132.2 | 11 | 1 | 100 |
| L-Alanine | 90.1 | 11 | 1 | 100 |
| L-Arginine | 175.2 | 11 | 1 | 100 |
| L-Asparagine | 133.1 | 11 | 1 | 100 |
| L-Aspartic acid | 134.1 | 11 | 1 | 100 |
| L-Cystine | 241.2 | 11 | 1 | 100 |
| L-Glutamic acid | 148.1 | 11 | 1 | 100 |
| L-Glutamine | 147.2 | 11 | 1 | 100 |
| L-Histidine | 156.1 | 11 | 1 | 100 |
| L-Lysine | 147.2 | 11 | 1 | 100 |
| L-Methionine | 150.1 | 11 | 1 | 100 |
| L-Phenylalanine | 166.2 | 11 | 1 | 100 |
| L-Proline | 116.2 | 11 | 1 | 100 |
| L-Serine | 106.1 | 11 | 1 | 100 |
| L-Threonine | 120.2 | 11 | 1 | 100 |
| L-Tryptophan | 205.3 | 11 | 1 | 100 |
| L-Tyrosine | 182.2 | 11 | 1 | 100 |
| L-Valine | 118.1 | 11 | 1 | 100 |

**Supplementary table 6. Single ion monitoring parameters.**

| Parameter | Value |
| --- | --- |
| Scan Rate (Hz) | 2.5 |
| Gas Temperature (°C) | 350 |
| Gas Flow (L/min) | 7 |
| Nebulizer (PSI) | 25 |
| Sheath Temperature (°C) | 400 |
| Sheath Gas Flow (L/min) | 12 |
| Capillary Voltage (V) | Positive Set Point: 4000, Negative Set Point: 4000 |
| Nozzle Voltage (V) | Positive Set Point: 0, Negative Set Point: 2000 |

**Supplementary table 7. Mass spectrometer parameters.**
